# Supplementary material for: Advanced Mass Spectrometric Techniques for the Comprehensive Study of Synthesized Silicon-Based Silyl Organic Compounds: Identifying Fragmentation Pathways and Characterization
Source: Materials (Basel). 2023 May 6;16(9):3563. doi: 10.3390/ma16093563 (PMC10179955; doi:10.3390/ma16093563)
Supplement: Supplementary file 1 [file materials-16-03563-s001.zip › materials-2369911-supplementary.pdf]

Advanced Mass Spectrometric Techniques for the Comprehensive Study of Synthesized Silicon-Based Silyl Organic Compounds: Identifying Fragmentation Pathways and Characterization

Agnieszka Rogowska [1,2], Małgorzata Szultka-Młyńska [2,*], Basem Kanawati [3], Paweł Pomastowski [1], Adrian Arendowski [1], Adrian Gołębiowski [1,2], Phillipe Schmitt-Kopplin [3], Marta Fordymacka [4], Jarosław Sukiennik [4], Julia Krzywik [4] and Bogusław Buszewski [1,2]

[1] Centre for Modern Interdisciplinary Technologies, Nicolaus Copernicus University in Torun, Wileńska 4, 87-100 Torun, Poland; aga4356@wp.pl (A.R.); pomastowski.pawel@gmail.com (P.P.); aarendowski@umk.pl (A.A.); adrian.golebiowski@doktorant.umk.pl (A.G.); bbusz@umk.pl (B.B.)
[2] Department of Environmental Chemistry and Bioanalytics, Faculty of Chemistry, Nicolaus Copernicus University in Torun, Gagarina 7, 87-100 Torun, Poland
[3] Research Unit Analytical BioGeoChemistry, Helmholtz Center Munich—German Research Center for Environmental Health, 85764 Neuherberg, Germany; basem.kanawati@helmholtz-munich.de (B.K.); schmitt.kopplin@helmholtz-munich.de (P.S.-K.)
[4] TriMen Chemicals Sp. z o.o., Al. Piłsudskiego 141, 92-318 Lodz, Poland; mfordymacka@trimen.pl (M.F.); jsukiennik@trimen.pl (J.S.); jkrzywik@trimen.pl (J.K.)
* Correspondence: mszultka@umk.pl

NMR spectra of synthesized compounds (Supplementary Figure S1)

1-O-(Trimethylsilyl)-2,3,4,6-tetra-O-acetyl-β-D-glucopyranose (compound 1)

^{1}H-NMR

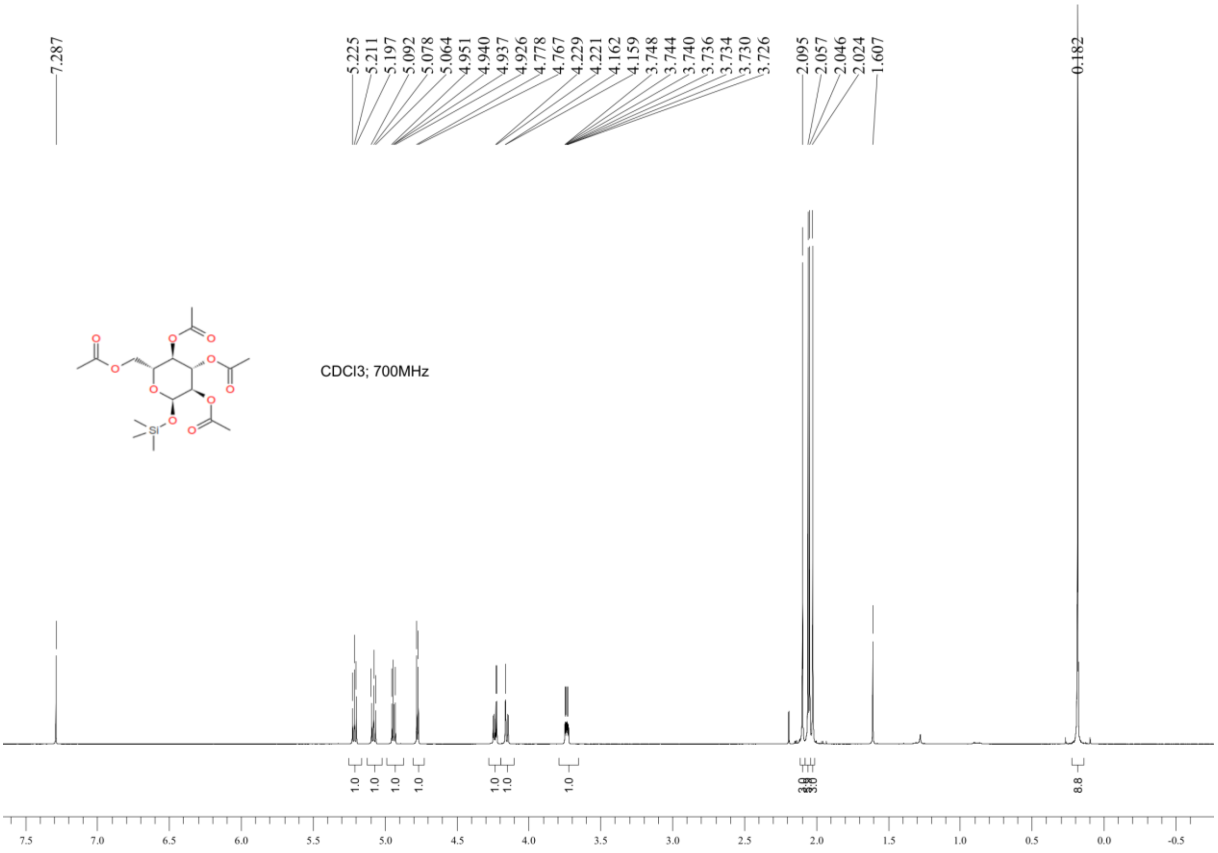

7.287
5.225
5.211
5.197
5.092
5.078
5.064
4.951
4.940
4.937
4.926
4.778
4.767
4.229
4.221
4.162
4.159
3.748
3.744
3.740
3.736
3.734
3.730
3.726
2.095
2.057
2.046
2.024
1.607
0.182
CDCl3; 700MHz
Si

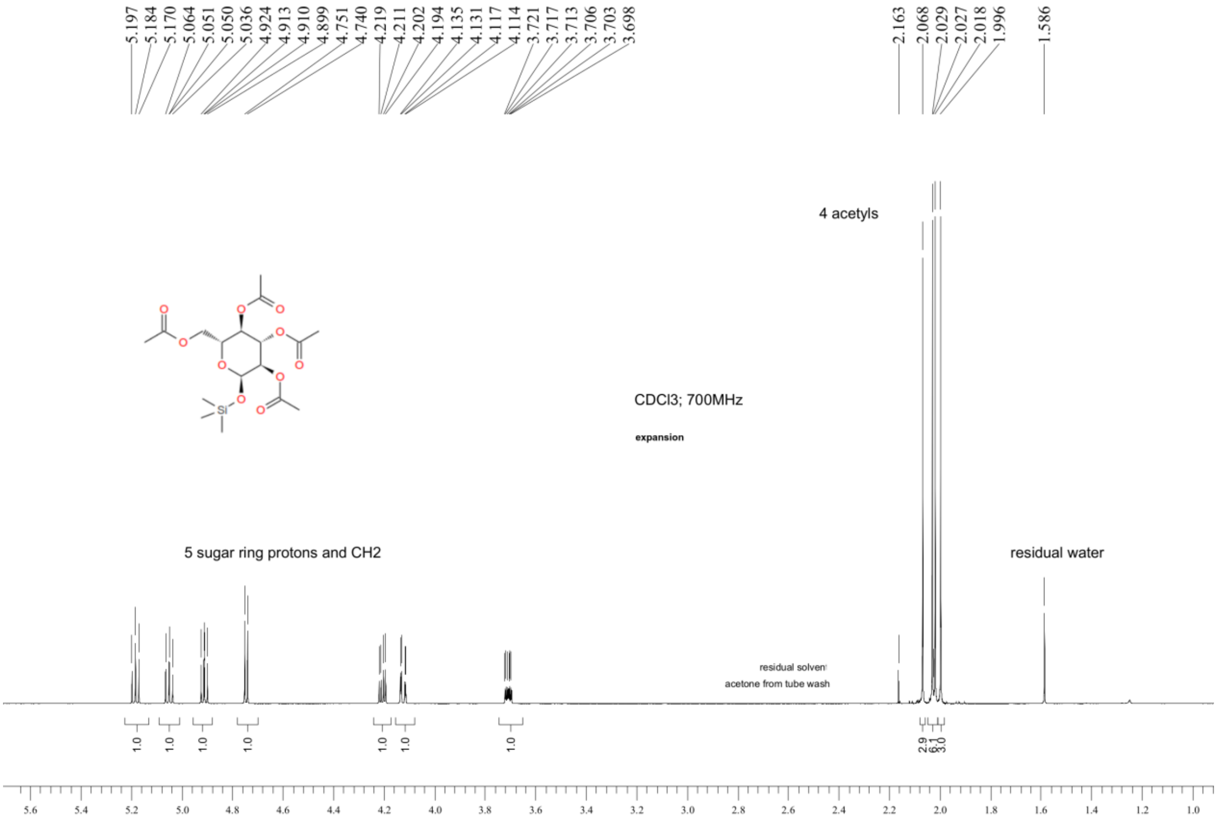

1- [(1,1-dimethylehtyl)diphenylsilyl]-1H-indole (compound 2)

1
H-NMR

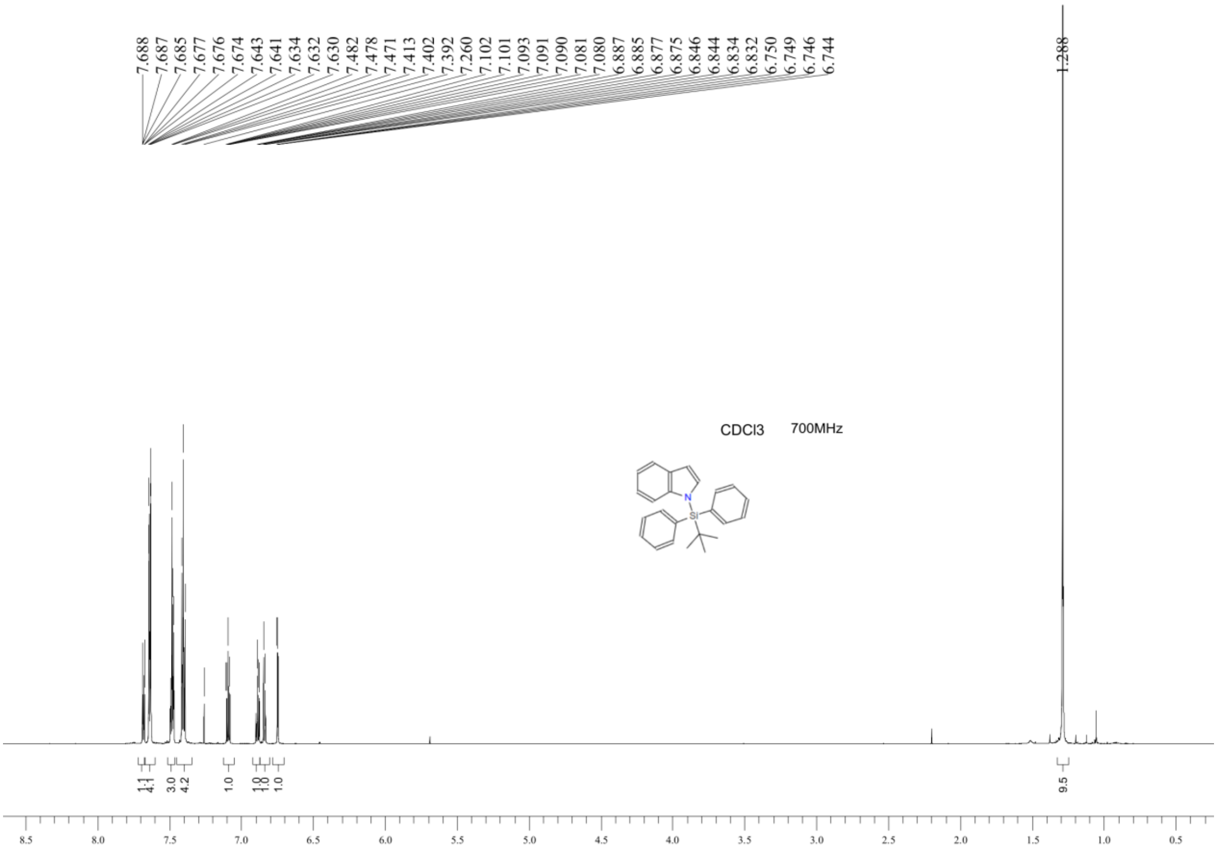
7.688 7.687 7.685 7.677 7.676 7.674 7.643 7.641 7.634 7.632 7.630 7.482 7.478 7.471 7.413 7.402 7.392 7.260 7.102 7.101 7.093 7.091 7.090 7.081 7.080 6.887 6.885 6.877 6.875 6.846 6.844 6.834 6.832 6.750 6.749 6.746 6.744
1.288
CDCl3 700MHz
N
Si
1.1 4.1 3.0 4.2 1.0 1.0 1.0 1.0
9.5
8.5 8.0 7.5 7.0 6.5 6.0 5.5 5.0 4.5 4.0 3.5 3.0 2.5 2.0 1.5 1.0 0.5

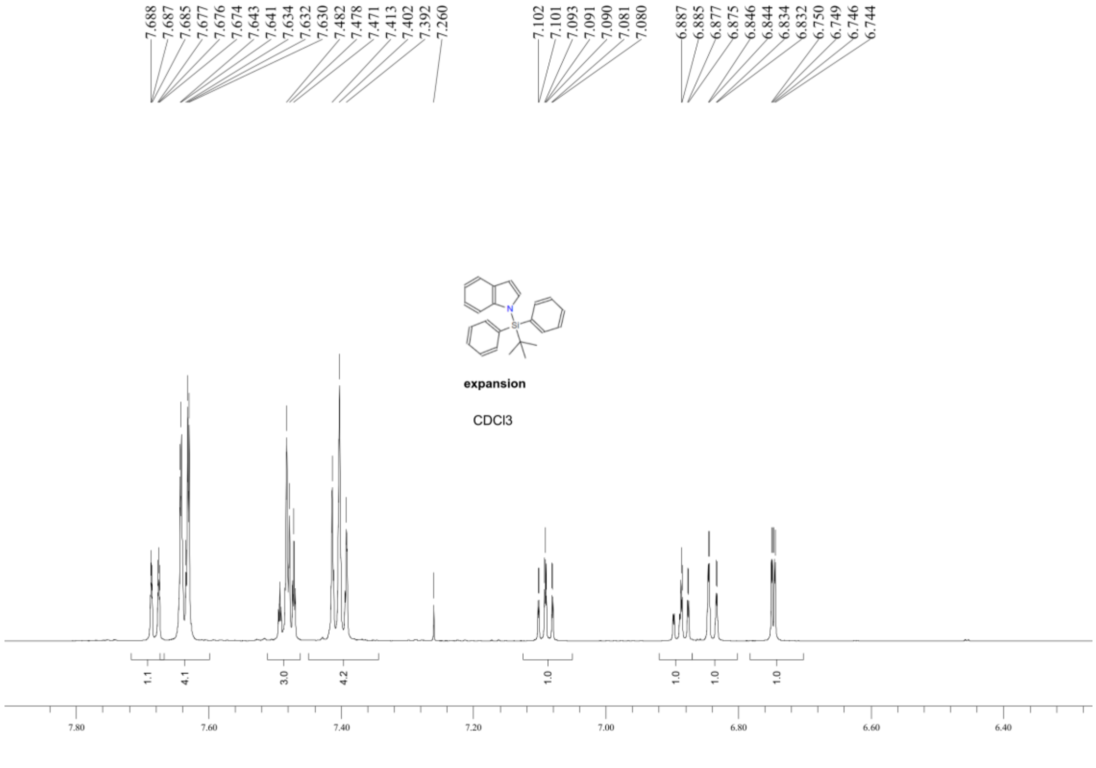
7.688 7.687 7.685 7.677 7.676 7.674 7.643 7.641 7.634 7.632 7.630 7.482 7.478 7.471 7.413 7.402 7.392 7.260
7.102 7.101 7.093 7.091 7.090 7.081 7.080
6.887 6.885 6.877 6.875 6.846 6.844 6.834 6.832 6.750 6.749 6.746 6.744
N
Si
expansion
CDCl3
1.1 4.1 3.0 4.2 1.0 1.0 1.0 1.0
7.80 7.60 7.40 7.20 7.00 6.80 6.60 6.40

O-tert-butyldiphenylsilyl-(3- hydroxypropyl)oleate (compound 3)

1
H-NMR

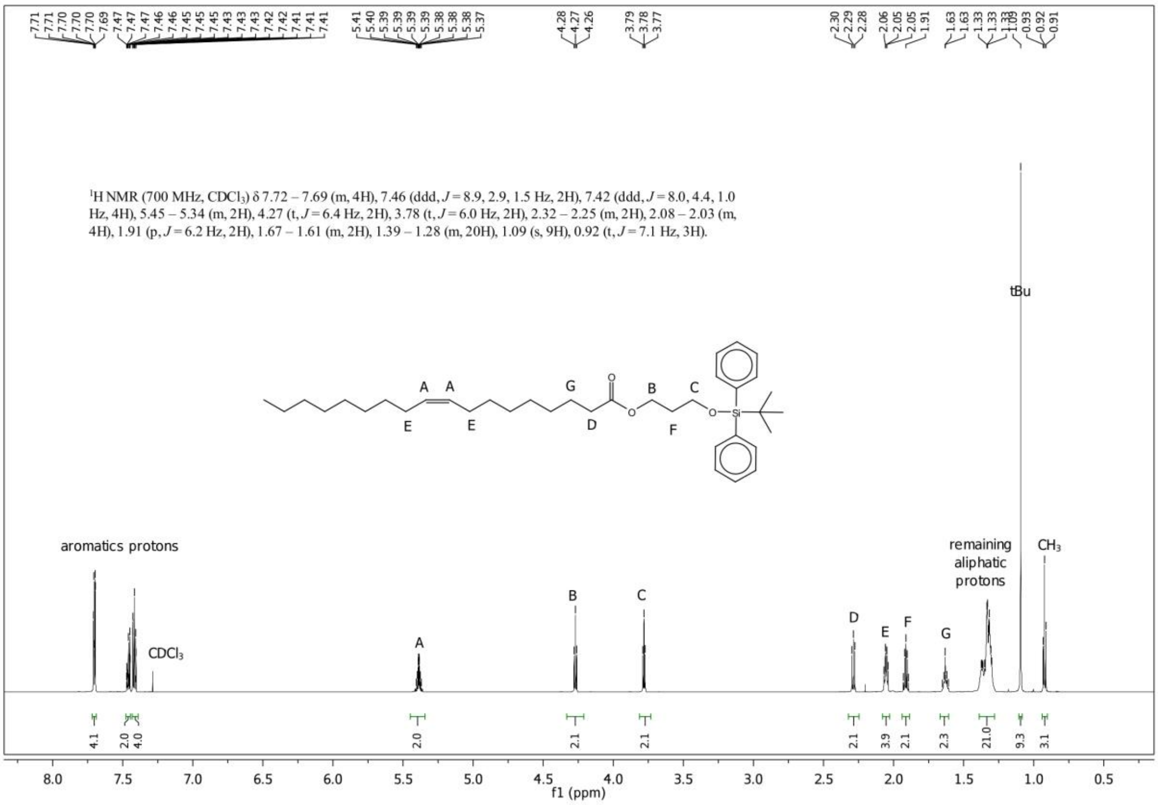

1-O-tert-Butyldiphenylsilyl-myo-inositol (compound 4)

^{1}H-NMR

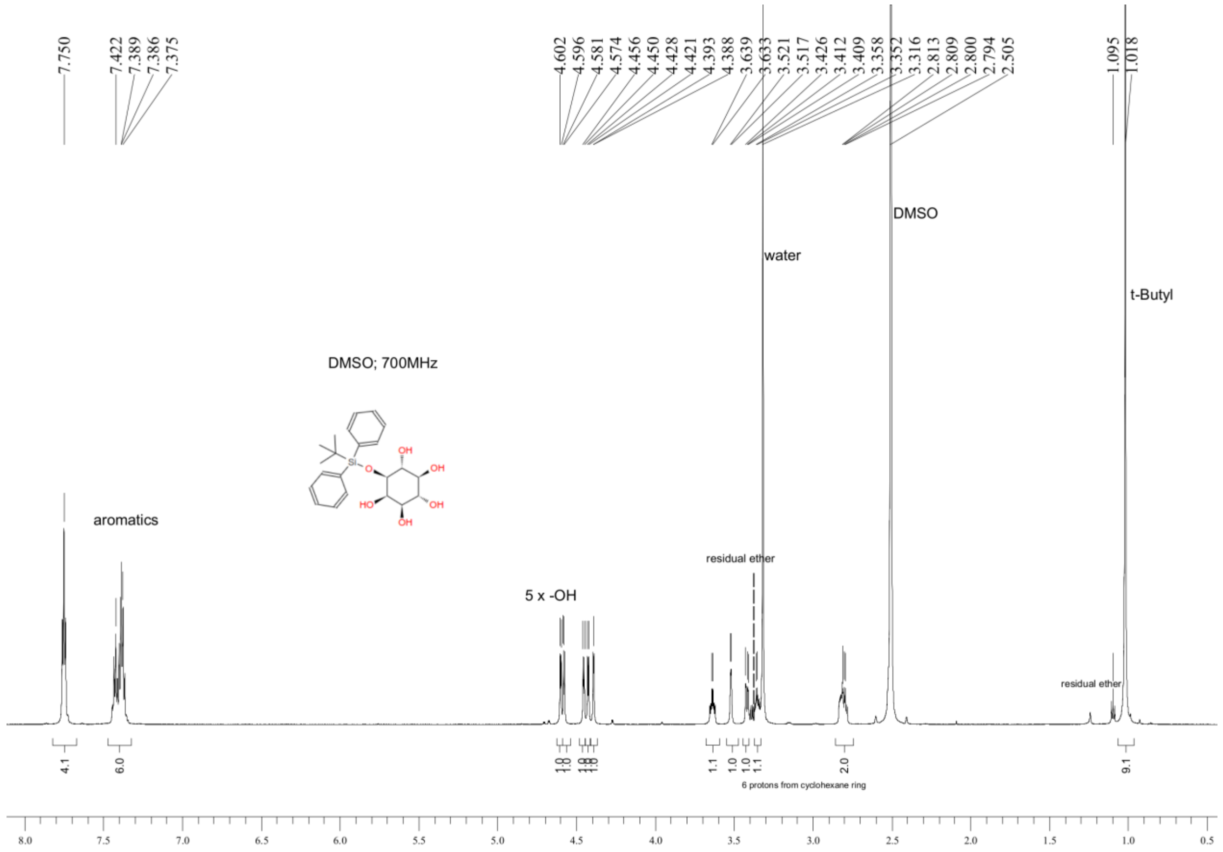

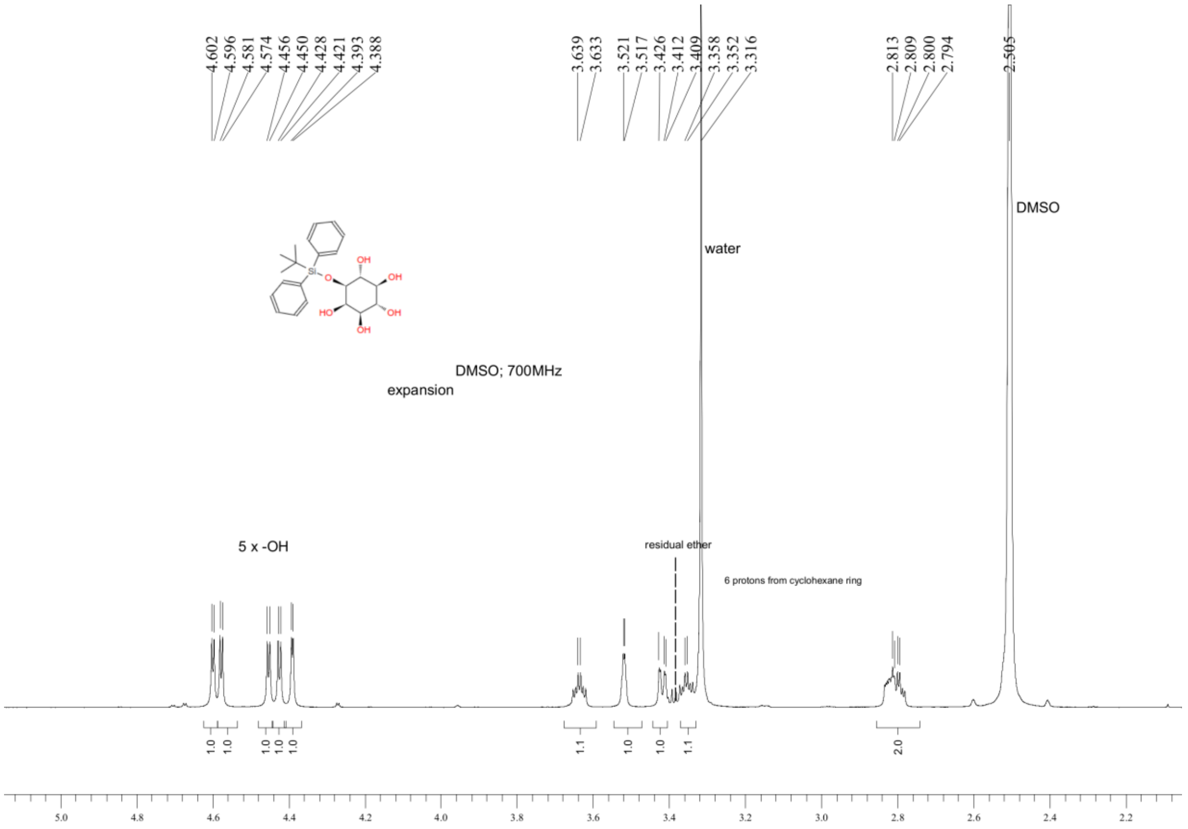

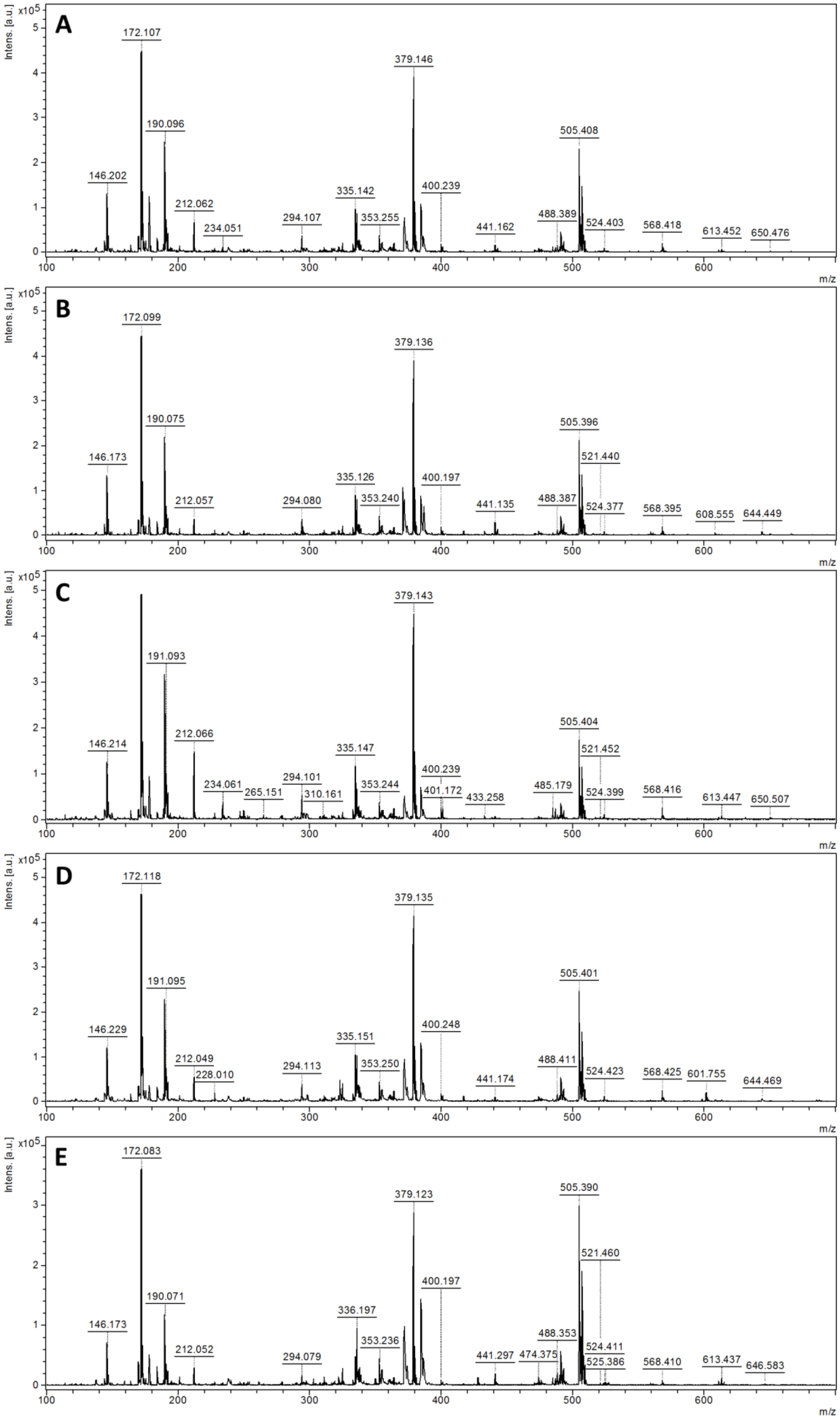
A
172.107
379.146
190.096
505.408
146.202
335.142
400.239
212.062
234.051
294.107
353.255
441.162
488.389
524.403
568.418
613.452
650.476
B
172.099
379.136
190.075
505.396
146.173
521.440
335.126
400.197
353.240
212.057
441.135
488.387
524.377
294.080
568.395
608.555
644.449
C
379.143
191.093
212.066
505.404
521.452
146.214
335.147
400.239
234.061
294.101
353.244
401.172
265.151
310.161
433.258
485.179
524.399
568.416
613.447
650.507
D
172.118
379.135
191.095
505.401
146.229
400.248
335.151
212.049
353.250
228.010
294.113
488.411
524.423
441.174
568.425
601.755
644.469
E
172.083
505.390
379.123
521.460
400.197
190.071
336.197
146.173
488.353
212.052
353.236
524.411
441.297
474.375
525.386
294.079
568.410
613.437
646.583
Intens. [a.u.]
x10^5
m/z

Supplementary Figure S2. The MALDI spectra of CHCA matrix (A) 1-O-(Trimethylsilyl)-2,3,4,6-tetra-O-acetyl-β-D-glucopyranose (B), 1-[(1,1-dimethylehtyl)diphenylsilyl]-1H-indole (C), O-tert-butyldiphenylsilyl-(3-hydroxypropyl)oleate (D) and 1-O-tert-Butyldiphenylsilyl-myo-inositol (E).

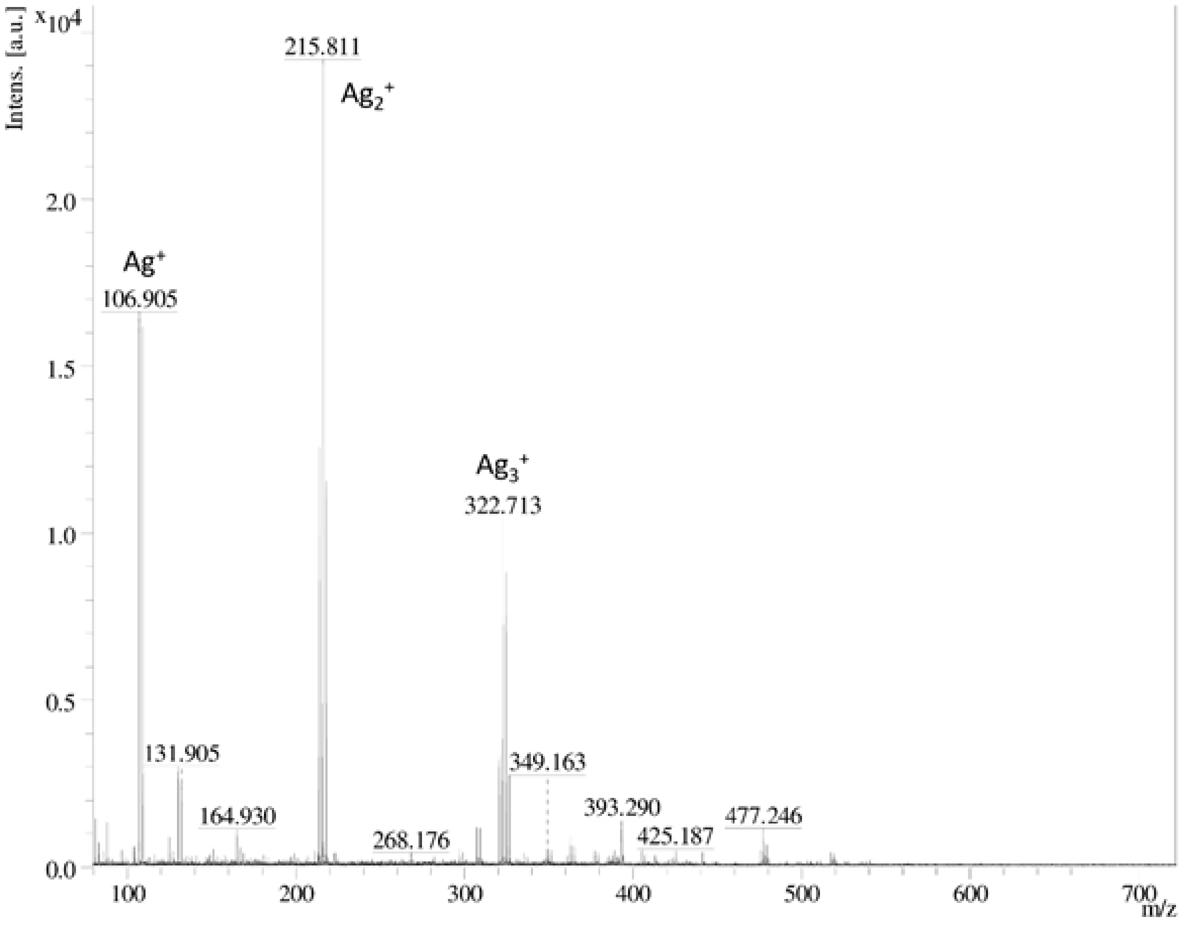

Supplementary Figure S3. The NALDI spectra of a SALDI target covered with silver nanoparticles.

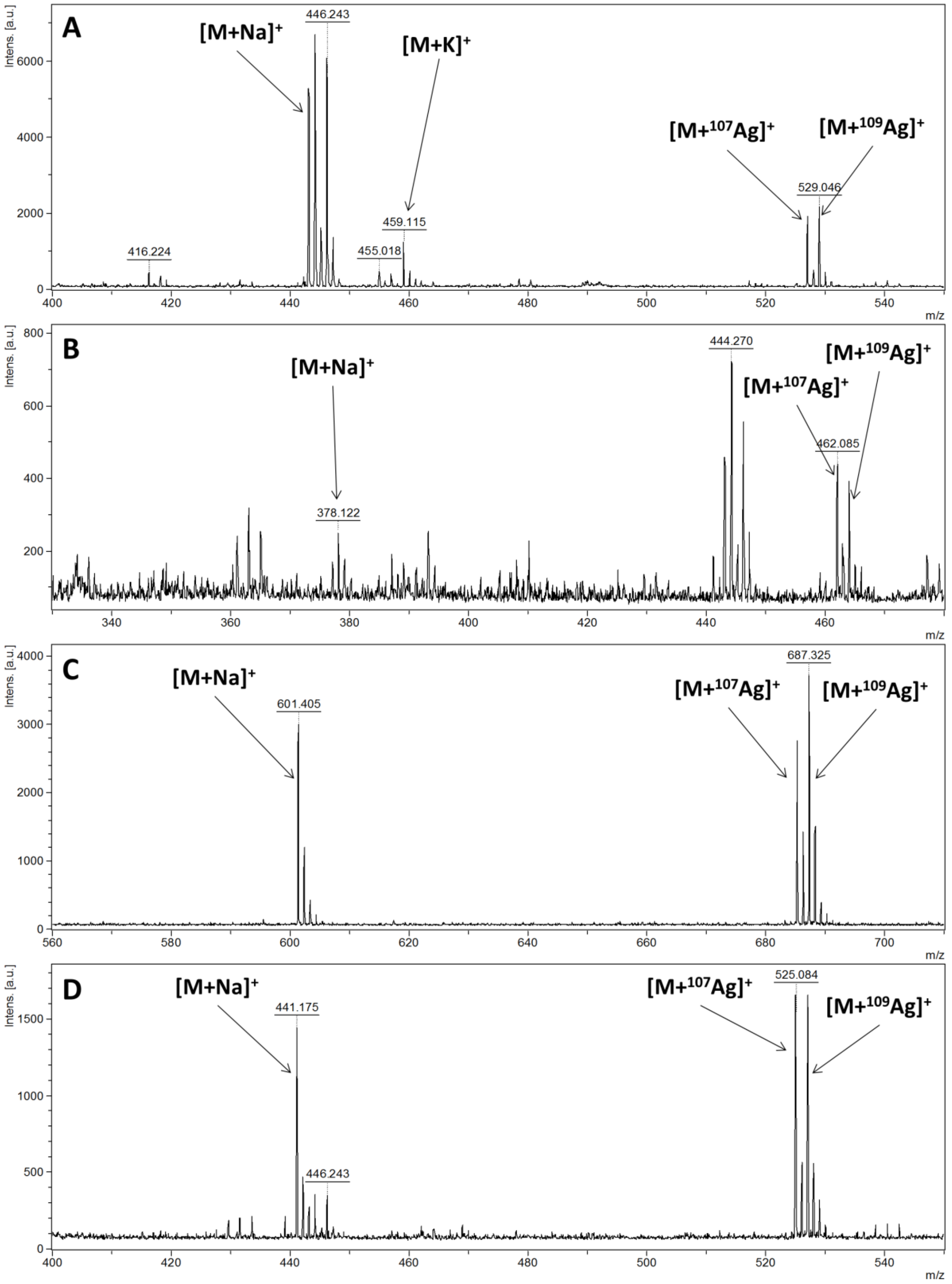

Supplementary Figure S4. The NALDI spectra of 1-O-(Trimethylsilyl)-2,3,4,6-tetra-O-acetyl-β-D-glucopyranose (A), 1-[(1,1-dimethylehtyl)diphenylsilyl]-1H-indole (B), O-tert-butyldiphenylsilyl-(3-hydroxypropyl)oleate (C) and 1-O-tert-Butyldiphenylsilyl-myo-inositol (D) with an enlarged area in which adducts are present.

Table S1. Selected procedures for the synthesis of similar compounds.

Compound	Precursor	Reagents and Conditions	Methods	Yield	Purity	Reference
1-O-(Trimethylsilyl)-2,3,4,6-tetra-O-acetyl-β-D-glucopyranose	2,3,4,6-tetra-O-acetyl-D-glucopyranose (34.8 g, 0.1 mol)	Chlorotrimethylsilane (15.2 mL, 0.12 mol), triethylamine (20.8 mL, 0.15 mol) in dichloromethane (130 mL) at room temperature	Nucleophilic substitution, Anomeric silylation	85%	Single NMR (500 MHz)	[1] J. Org. Chem. 1993, 58, 4175-4178
2-Acetylamino-1,3,4,6-tetra-O-(trimethylsilyl)-2-deoxy-α-D-glucopyranose	N-acetyl-D-glucosamine (1.0 g, 4.52 mmol)	Hexamethyldisiloxane (8.0 mL, 38.90 mmol), chlorotrimethylsilane (4.0 mL, 31.64 mmol) in pyridine (10 mL) at 353 K	Nucleophilic substitution, Protection of amino group via silylation	Not provided	Not provided	[2] Acta Cryst. 2013, 69, o917
1D-1-O-tert-Butyl-diphenyl-silyl-2,3,6-O-tris-(methoxymethylene)-myo-inositol 4,5-bis-(dibenzylphosphate)	Triol (10.2 g, 16.2 mmol) and ethyldiisopropylamine (DIPEA) (14.4 mL, 82.7 mmol) in DMF (30 mL), methyl chloromethyl ether (MOM-Cl) (4.8 mL, 63.2 mmol)	Reaction proceeded for 24 hours at 65˚C	Purification (hexane/acetone, 8:1)	Not provided	Colorless glass	[3] Acta Cryst. 2012, 68, o900
4-Bis(trimethylsilyl) indole	1-(Trimethylsilyl)indole, chlorotrimethylsilane, lithium metal, THE solution	Ultrasonic agitation for 4 hours at 5-10°C and overnight at 45°C	Evaporation of the solvent gave an off-white solid that was air sensitive and presumably contained lithium chloride and 8b. Without purification, this was directly oxidized using 1,4-benzoquinone in dichloromethane solution. Chromatography gave 1,4-	55%	Single 1H NMR	[4] J. Org. Chem. 1984, 49, 4409-4415

			bis(trimethylsilyl) indole (7d)			
Silicon Oleochemicals	Fatty acid esters with double bonds (e.g., methyl undec-10-enoate, methyl linoleate, methyl α-linolenate, ethyl oleate), chloro- or alkoxyhydrosilanes, H_2PtCl_6 catalyst	1.98 g (10.0 mmol) methyl undec-10-enoate and 10.0 mmolhydrosilane, 8 ml cyclohexane 0.0410 g (0.10 mmol) H_2PtCl, dissolved in 8 ml propylenecarbonate, 10.0 mmol unsaturated fatty acid ester and 18.0 mmol hydrosilane were mixed under an argon atmosphere	Hydrosilylation using Speier's catalyst in bi- and single-phasic systems	4-77%	1H-NMR (400MHz)	[5] Eur. J. Lipid Sci. Technol. 2002, 104, 161–166

1. Allevi, P.; Anastasia, M.; Ciuffreda, P.; Bigatti, E.; Macdonald, P. Stereoselective glucosidation of podophyllum lignans. A new simple synthesis of etoposide. *J. Org. Chem.* **1993**, *58*, 4175-4178, doi: 10.1021/jo00067a071.

2. Cheng, Z.D.; Cui, Y.L.; Mao, J.W. 2-Acetylamino-1,3,4,6-tetra-O-(trimethylsilyl)-2-deoxy-a-D-glucopyranose. *Acta Cryst.* **2013**, *69*, o917, doi: 10.1107/S160053681301266X.

3. Anderson, R.J.; Gainsford, G.J. 1D-1-O-tert-Butyldiphenylsilyl-2,3,6-Otris(methoxymethylene)-myo-inositol 4,5-bis(dibenzylphosphate). *Acta Cryst.* **2012**, *68*, o900, doi: 10.1107/S1600536812008069.

4. Dauzonne, D.; O'Neil, I.A.; Renaud, A. Preparation and reactions of 4-(trimethylsilyl)indole. *J. Org. Chem.* **1984**, *49*, 4409-4415, doi: 10.1021/jo00197a016.

5. Behr, A.; Naendrup, F.; Obst, D. The synthesis of silicon oleochemicals by hydrosilylation of unsaturated fatty acid derivatives. *Eur. J. Lipid Sci. Technol.* **2002**, *104*, 161–166, doi: 10.1002/1438-9312(200203)104:3<161::AID-EJLT161>3.0.CO;2-N.
